# Supplementary material for: Impact of protected areas on deforestation in Madagascar from 2000 to 2023: A pre-analysis plan
Source: PLoS One. 2026 Feb 9;21(2):e0342093. doi: 10.1371/journal.pone.0342093 (PMC12885278; doi:10.1371/journal.pone.0342093)
Supplement: S1 Table — It summarizes the different variables used in the analysis. The selected articles applied matching method to assess the impact of conservation policies and addressed similar research questions. The table includes 11 studies, including 6 carried out in Madagascar. While not an exhaustive review of impact evaluations of conservation programs in Madagascar, it provides an overview of the variables taken into account to find the protected areas counterfactual given the main drivers of deforestation. (DOCX) [file pone.0342093.s001.docx]

# **Supporting information**

**S1 Table**

| Reference | Study coverage | Matching | Outcome variable | Treatment variable | Matching variable | Estimation Covariables | Analysis unit | Buffer | Tree Cover Density (TCD) | Robustness test |
| --- | --- | --- | --- | --- | --- | --- | --- | --- | --- | --- |
| A forest loss report card for the world's protected areas [1] | Global  (2001-2018) | Yes  1-k Coarsened exact matching | Deforestation rate in PAs | Protected Area | Slope  Elevation  Forest cover  Travel time to nearest densely populated area  Average population density within a 20 km radius  Country  Ecoregion  Main cause of forest cover loss | The richness of threatened and non-threatened vertebrate species,  The year in which the PAs were established,  Surface  GDP/capita in PPP | 1km* 1km | 10 km excluded | > 30 % | Rosenbaum test |
| Estimating the Counterfactual Impact of Conservation Programs on Land Cover Outcomes: The Role of Matching and Panel Regression Techniques [2] | Northeastern Ecuador (2004-2013) and European Russia  (1995-2010) | Yes, matching with fixed effect | Northeastern Ecuado (PES)r= Percent change in forest cover  European Russia (PA)= Forest disturbance for harvesting timber | Payment for ecosystem services (PES) and strict protected area  (Category I IUCN) | PES:  Observable covariates:  Parcel size  Distance of the parcels to roads  Distance of the parcels to population centers  Distance of the parcels to oil wells  Distance of the parcels to navigable rivers  Unobservable variates:  Environmental motivations  Parcel-level agricultural productivity  Protected area:  Observable variables  Slope  elevation  Distance to the forest edge  Distance from closest town  Distance to Moscow  Distance to the closest road | Same as matching variables | 30m* 30m | Not mentioned | Not mentioned | Not mentioned |
| How Effective Are Biodiversity Conservation Payments in Mexico? [3] | Marqués de Comillas et Benemérito de las Américas (2007-2013) | Yes, matching Mahalanobis distance | Avoided deforestation | Payment for ecosystem Services | Forest cover in 2007  Elevation  Slope  Risk of deforestation  ejido size  Distance to nearest town  Size of conserved forests  Percentage of working population in 2005  Marginality index in 2005  Year after the creation of the ejido | Not mentioned | 10ha | Not mentioned | Not mentioned | *DiD with to control  *Addition of covariates (total population, percentage of population having completed high school, number of right-handed people), |
| Titling community land to prevent deforestation: An evaluation of a best-case program in Morona-Santiago, Ecuador [4] | Morona- Santiago, Ecuador  2000-2012 | Yes | Change in forest cover | Title issuance program | Elevation  Slope  Population density in 5km² (n-1)  Loss of forest cover in an area of 5km² (n-1)  percentage of forest cover (n-1)  Distance from main road  Distance to power supply  Distance from a river  Distance from disturbed land classification  Shuar indigenous lands  Protected area status | Not mentioned | 27,984 0.87 km² of land | Not mentioned | Not mentioned | Analysis of areas classified by USAID as uninhabited by the Shuar community during the PSUR program |
| How much deforestation do protected areas avoid in tropical Andean landscapes? [5] | Ecuador’s tropical Andean forest (1990-2008) | Yes, matching using propensity scores | Avoided deforestation | Protected area created between 1990 and 2008 | Elevation  Slope  Soil productivity  Distance from roads  Distance to towns  Distance from rivers | Not mentioned | 30m* 30m | 10km | Not mentioned | Not mentioned |
| ‍‍‍‍‍‍‍‍‍‍‍‍‍‍‍‍‍‍‍‍‍‍‍‍‍‍‍‍‍‍‍The impact of Protected Areas on deforestation? An exploration of the economic and political channels for Madagascar’s rainforests (2001- 2012) [6] | Madagascar (2001-2012) | Yes, Genetic matching | Deforestation rate in commune i for year t | PA and NPA from the eastern ecoregion that was officially included in the SAPM in 2012 | Socio-economic variables  Travelling time to nearest town – rainy season (hours)  Population in agricultural sector (%)  Irrigated rice paddy per inhabitant (%)  Poor people (%)  Destitute people (%)  Police  Population commune 2001  Population district (Average 2001-12)  Biophysical data  Average slope (%)  Average elevation (meters) | Not mentioned | 30m * 30m | Not mentioned | >78% | Use of different matching methods:  Mahalanobis, Propensity score, equal weights, 2 nearest neighbor |
| Effectiveness of community forest management at reducing deforestation in Madagascar [7] | Madagascar  (2000-2010) | Yes  Mahalanobis Matching | Whether or not the pixel remained deforested in 2010 | Community forest management | Land suitable for agriculture  Land unsuitable for farming  Land suitable for irrigated agriculture  Slope  Elevation  Distance from most recent deforestation  Distance from forest edge  Distance from a village  Distance from a main road  Distance from a cart path  Travel time to an urban center  Population density in 2003 | Not mentioned | Not mentioned | Not mentioned | Not mentioned | Rosenbaum sensitivity test for unobservable covariates |
| Contrasting spatial and temporal trends of protected area effectiveness in mitigating deforestation in Madagascar [8] | Madagascar  (1990-2000  2000-2010) | Yes Mahalanobis distance | Loss of forest cover | Protected Area | Elevation  Slope  Distance from a large city (population > 100,000 in 1993)  Distance from main road  Distance from the river.  Annual precipitation | The same | 30m* 30m | Not mentioned | Not mentioned | Not mentioned |
| ‍‍‍‍‍‍‍‍‍‍‍‍‍‍‍‍‍‍‍‍‍‍‍‍‍‍‍‍‍‍‍Rain, forest and farmers: Evidence of drought induced deforestation in Madagascar and its consequence for biodiversity conservation [9] | Madagascar (2000-2013) | Yes, Hainmueller matching | Forest cover loss | Protected area created in 2000 | Average elevation  Average slope  Long-term average mevel of rainfall  Long-term average temperature  Area of forest in 1990  Area of forest in 1990 in a 0.5 degree buffer around the cell’s centroïd | Not mentioned | 10km *10km | Not mentioned | Specific for each ecoregion but not mentioned | Not mentioned |
| What constitutes a useful measure of protected area effectiveness? A case study of management inputs and protected area impacts in Madagascar [10] | Madagascar  (2005-2010) | Yes | Avoided deforestation | State governed Protected Area | Distance from forest edge  Elevation  Slope  Annual precipitation  Distance from nearest town  Distance from rivers  Distance from roads  Shape and size of protected area | Not mentioned | 1ha | Not mentioned | Not mentioned | Not mentioned |
| On track to achieve no net loss of forest at Madagascar's biggest mine [11] | Madagascar (2001-2019) | Yes  1:1 nearsest neighbor (Mahalanobis distance 1 s.d) | Annual deforestation rate | Protected Area | Elevation  Slope  Distance from main road  Distance from forest edge  Distance from last deforested area | Annual precipitation  Distance from the river  Distance from the cart track  Distance from the village  Population density | 30m*30m | 10km  excluded | >75 % | Testing 116 other specifications different from the main model :  different distance measures (Mahalanobis, standard propensity score, propensity score using Randomforest,  test different standard deviation calibrations (0.25, 0.5 and 1)  different numbers of control units 1:1, 1:5, 1:10  addition of covariates |

Source : Authors (2025)

# References

1. Wolf C, Levi T, Ripple WJ, Zárrate-Charry DA, Betts MG. A forest loss report card for the world’s protected areas. Nat Ecol Evol. 2021;5: 520–529. doi:10.1038/s41559-021-01389-0

2. Jones KW, Lewis DJ. Estimating the Counterfactual Impact of Conservation Programs on Land Cover Outcomes: The Role of Matching and Panel Regression Techniques. PLOS ONE. 2015;10: e0141380. doi:10.1371/journal.pone.0141380

3. Costedoat S, Corbera E, Ezzine-de-Blas D, Honey-Rosés J, Baylis K, Castillo-Santiago MA. How Effective Are Biodiversity Conservation Payments in Mexico? PLOS ONE. 2015;10: e0119881. doi:10.1371/journal.pone.0119881

4. Buntaine MT, Hamilton SE, Millones M. Titling community land to prevent deforestation: An evaluation of a best-case program in Morona-Santiago, Ecuador. Global Environmental Change. 2015;33: 32–43. doi:10.1016/j.gloenvcha.2015.04.001

5. Cuenca P, Arriagada R, Echeverría C. How much deforestation do protected areas avoid in tropical Andean landscapes? Environmental Science & Policy. 2016;56: 56–66. doi:10.1016/j.envsci.2015.10.014

6. Desbureaux S, Aubert S, Brimont L, Karsenty A, Lohanivo AC, Rakotondrabe M, et al. The impact of Protected Areas on Deforestation? An Exploration of the Economic and Political Channels for Madagascar’s Rainforests (2001-12). Working Papers. HAL; 2015 July. Report No.: hal-01176860. Available: https://ideas.repec.org/p/hal/wpaper/hal-01176860.html

7. Rasolofoson RA, Ferraro PJ, Jenkins CN, Jones JPG. Effectiveness of Community Forest Management at reducing deforestation in Madagascar. Biological Conservation. 2015;184: 271–277. doi:10.1016/j.biocon.2015.01.027

8. Eklund J, Blanchet FG, Nyman J, Rocha R, Virtanen T, Cabeza M. Contrasting spatial and temporal trends of protected area effectiveness in mitigating deforestation in Madagascar. Biological Conservation. 2016;203: 290–297. doi:http://dx.doi.org/10.1016/j.biocon.2016.09.033

9. Desbureaux S, Damania R. Rain, forests and farmers: Evidence of drought induced deforestation in Madagascar and its consequences for biodiversity conservation. Biological Conservation. 2018;221: 357–364. doi:10.1016/j.biocon.2018.03.005

10. Eklund J, Coad L, Geldmann J, Cabeza M. What constitutes a useful measure of protected area effectiveness? A case study of management inputs and protected area impacts in Madagascar. Conservat Sci and Prac. 2019;1: e107. doi:10.1111/csp2.107

11. Devenish K, Desbureaux S, Willcock S, Jones JPG. On track to achieve no net loss of forest at Madagascar’s biggest mine. Nat Sustain. 2022;5: 498–508. doi:10.1038/s41893-022-00850-7
