# Supplementary material for: Impact of protected areas on deforestation in Madagascar from 2000 to 2023: A pre-analysis plan
Source: PLoS One. 2026 Feb 9;21(2):e0342093. doi: 10.1371/journal.pone.0342093 (PMC12885278; doi:10.1371/journal.pone.0342093)
Supplement: Attachment — Submitted filename: Response to Reviewers.pdf [file pone.0342093.s002.pdf]

Impact of protected areas on deforestation in Madagascar from 2000 to 2023 : A pre-analysis plan

RESPONSE TO EDITOR AND REVIEWERS

Dear Dr Daniel de Paiva Silva,

We sincerely thank you for agreeing to review our manuscript entitled « Impact of Protected Areas on Deforestation in Madagascar from 2000 to 2023 : A pre-analysis plan ».

We are grateful for the time and effort you devoted to evaluating our work, which reflects your interest in our research. We also greatly appreciate that you have given us the opportunity to resubmit our paper, despite it not fully meeting PLOS ONE's publication criteria. We have carefully considered your comments, as well as those from the reviewers, to improve our manuscript. All the comments and suggestions have been addressed, and the corresponding revisions are detailed below.

<u>Response to reviewers :</u>

Response to Reviewer #1

This report meets the criteria for the Registered Report Protocol publication format by describing the state of knowledge, research questions and methodology for the future development of a research proposal. The results of this research proposal will have an impact on the conservation of protected areas in tropical regions by providing insights into predicting the impacts of forest loss on protected areas. I have a few observations:

We sincerely thank you for your positive feedback and for your interest in our work.

L100-102. In this section, it is necessary to provide a brief background on the benefits of using Coarsened Exact Matching (CEM) and Difference-in-Differences (DiD)

The benefits of using CEM and DiD have been added from the line 141 to the line 154.

L110-128 This section should be part of the introduction or materials and methods.

The section about the pre-analysis plan objectives has been moved to the "Materials and methods' section" in line 183.

Response to Reviewer #2

1) Here, or in the Introduction to the main Registered Report, I recommend you discuss some of the challenges facing conservation and Protected Areas in Madagascar: paper parks, lack of funding, lack of income from tourism in the majority of PAs, corruption, insecurity (daholos etc.), lack of enforcement capacity. This is really important framing for your study, emphasizing why it is important to evaluate the effectiveness of PAs individually, so you can try and understand what factors are associated with PA successes or failure.

Thank you for this recommendation. We recognize that the introduction lacked sufficient contextualization. We have revised the pre-registered protocol accordingly and will continue refining it for the main Registered Report. In particular, as you suggested, we have highlighted Madagascar's specific conservation challenges, including governance issues, lack of funding and the widespread prevalence of poverty from line 52.

2) In the Introduction you suggest that the Hansen Global Forest Change data is better to use than the Harper et al forest cover maps for measuring forest cover change. You talk about the limitations of the Harper et al., data (which come from the Eklund et al., paper and not the

original source) but do not mention at all the known limitations of the GFC data and other global products. This is unbalanced and gives the impression that the Harper et al., data is 'wrong' and the Hansen GFC data is 'correct'. In my experience, this is not the case. I've used the Harper et al maps, the Vieilledent et al., 2018 (https://www.sciencedirect.com/science/article/pii/S0006320718301125), the Global Forest Change data (Hansen et al., 2013) and the more recent Tropical Moist Forests (Vancutsem et al., 2022) data for studying deforestation in Madagascar. Both the Hansen data and the TMF data seem to substantially over-estimate deforestation in Eastern Madagascar, showing massive deforestation between the eastern edge of the CAZ and the east coast. However, the forest cover maps of Harper et al., suggest that these areas were not forested at baseline in 2000.

Personally, I prefer the approach taken by Vielledent et al., 2018. They combined the Harper et al., maps with the GFC data, masking the GFC forest loss data to the map of forest cover in 2000 from Harper et al.,. In the Eastern rainforests, this masking process seems to remove many false positives, where deforestation is detected in places which have not been forested for decades before 2000. I have used the same method (masking global deforestation datasets to the national map for the year 2000) in two previous impact evaluation studies of deforestation in Madagascar. I think this is justifiable for the following reasons. The GFC data is a global dataset so the classification algorithm has to be broad enough to capture deforestation in all the different forest ecosystems on earth. As such, it sacrifices local accuracy for global generalisability. The GFC is also known to be less accurate in capturing dynamics of dry forests. In contrast, the Harper et al., maps were designed and tailored to capture the specific ecological characteristics of Madagascar's forests. However, this dataset doesn't have a yearly time series and wasn't explicitly designed to capture changes. In my opinion combining the two datasets gives the most accurate measure of deforestation on land which was actually forested at the baseline in 2000.

I would strongly recommend you mask the Hansen et al data to the 2000 forest cover map from Harper et al., and take a look. If there are any forested areas you are personally familiar with, compare the deforestation shown in these areas with and without masking.

Previous studies have shown that the choice of data to measure outcomes can have a strong effect on the results of impact evaluations and I've seen how much of a difference there is between the global deforestation maps and the Harper et al., map for 2000. If you choose to continue using the Hansen et al., 2013 data for your analysis and I am asked to review the Registered Report, I would want to see additional results using the Hansen data masked to the 2000 forest cover map as a robustness check. Without this I would have doubts about any significant results.

Thank you for your comments, which are very relevant to our study. Indeed, we had not clearly explained how we intend to use the data from Hansen et al. (2013). We will use the 2000 forest cover data from Hansen et al. (2013), incorporating average tree canopy density thresholds specific to each simplified forest type, as determined by study conducted by the World Resources Institutes (WRI) team in Madagascar, in collaboration with other colleagues in 2023. This study, entitled "Tree Canopy Density for Improved Forest Cover Estimation in Protected Areas of Madagascar" (Rafanoharana et al. 2023), aimed to identify tree canopy density (TCD) thresholds in Madagascar's protected areas. In addition, the local team works to assess the reliability of Hansen et al. (2013) forest cover data and its updated versions, and ensures that local stakeholders use these data appropriately. Rafanoharana et al.'s paper builds upon the work of Vielledent et al.(2018), who developed a new forest cover map for Madagascar by combining the forest cover data of Harper et al. (2007) with the deforestation data form Hansen et al. (2013). However, in that study, the authors applied the global tree canopy density threshold defined by Hansen et al. (2013) to distinguish forest from non-forest areas. Rafanoharana et al. (2023), in turn, used the updated map from Vieilledent et al. (2018) to develop TCD thresholds specifically adapted to protected areas.

As noted by Rafanoharana et al. (2023), Madagascar hosts a wide diversity of forest types, and applying a standard tree canopy density threshold leads to inaccurate estimates of forest typologies. In their study, Rafanoharana et al. (2023) identified specific tree canopy density thresholds for 111 protected areas listed in the 2015 database of Madagascar's Protected Areas System. However, they found that 20 of these protected areas consisted of non-contiguous parcels, which required identifying a specific threshold for each parcel.

In our approach, we will use the average tree canopy density thresholds of protected areas belonging to the same simplified forest type, as identified by Rafanoharana et al. (2023), and apply this average to the entire biome.

Moreover, we are aware of the limitations of the Hansen et al. (2013) data, particularly regarding their forest definition and their use of a standard tree canopy density threshold. This is why we rely on the specific thresholds proposed by Rafanoharana et al. (2023), while maintaining the use of the data from Hansen et al. (2013) to benefit from its temporal granularity.

Finally, masking the Hansen et al. (2013) deforestation data using the Harper et al. (2007) forest cover map for the year 2000 is a great idea. We have added your suggestion to the list of robustness checks in the pre-analysis plan in the line 605.

3) I don't think it's necessary to run a robustness check using cells of 5km2, I think that's too large and doesn't correspond to the scale of land use decision making. Personally, I also would use 1ha as the main scale of analysis. For me, alignment with the scale of land use decision making is more important that alignment with the scale of all covariates. At a scale of 1ha, ~10 neighbouring units will fall within the same 1km2 pixel in the population density and accessibility data. Therefore, all these 10 units will have the same value for these variables. I think that's okay because in reality, the true values of these variables will differ little within 1km2, especially for the distance variable (which will vary by up to 1km). While I personally would use 1ha as the main scale of analysis and 1km as a robustness check, I will leave it to

your discretion which one you use as the main scale. I am happy as long as you do run the analysis at 1 ha resolution (as main or additional).

Indeed, for our study, the choice of analysis unit size was the subject of careful consideration. As noted by Avelino et al. (2016), the scale of explanatory variables and the size of observation units can influence treatment effect estimates and introduce a bias known as the *Modifiable Areal Unit Problem* (MAUP), especially in conservation. In Madagascar, subsistence agriculture is the primary driver of deforestation, and agricultural plots are generally smaller than 1 ha (MAEP & FAO, 2007). However, most of the explanatory variables we will use have a resolution of 1 km^2, which underlies our initial choice.

Nevertheless, we will also conduct a robustness test using 1 ha analysis units to assess the impact of this scale effect on our results.

5) I have to admit that I'm confused about your leakage analysis. I am not familiar with the Butts paper but you need to make sure your method is sufficiently clear here so that readers do not need to cross-check with another paper. I'll try to explain what is causing my confusion to help you with this:

Lines 400-402: 'These new treatment zones will be matched with remote control zones to obtain the magnitude of spillover and leakage effects.'

This suggests that you will run the leakage analysis separately to the main within-PA analysis, setting units within the buffer ring zones as treated (and therefore excluding units within the PAs), matching to similar controls and running the regressions. This is again suggested in Lines 408 when you refer to buffer zone units as the 'treatment unit'. And in Lines 433 – 435.

If this is correct you need to make it really clear at the start of the 'Difference-in-differences with spillover and leakage effects' section that this is an additional, separate analysis to the main within-PA analysis. It is currently not clear and is confusing. In this additional analysis units within concentric buffer zones outside the PA will be assigned as treated, with treatment

occurring in the year the adjacent PA was protected. You can also state in the 'Treatment Assignment' section, that you will run two analyses, the first using units from within PAs as treated, and the second using units from a 20km buffer zone around each PA as treated. Additionally, to make it clear that this is a separate analysis, you should also include the regression equation for the main within-PA analysis in the 'Basic model of Difference-in-differences'. This will make it clear to readers that the two regressions are different (because the basic model doesn't include the Ring parameter), therefore this is two separate analyses. Also, regarding the leakage regression equation shown in Line 411, I'm not convinced about the (1-D) x Ring. If my interpretation is correct, we are interested in the interaction between the ring distance and D = 1, to estimate the effect of a treated buffer zone unit being further from the boundary of a PA (but still within the 20km buffer) after the PA is protected. But 1-1 = 0. So by including (1-D) x Ring aren't you completely removing the Ring term for the treated units you are interested in?

If I am wrong, and the two analyses are not separate, then you need to explain better how treatment is assigned

We recognize that our description of the spillover and leakage methodology was insufficiently clear. In fact, Butts (2023) does not recommend running a fully separate DiD analysis; rather, spillover and leakage effects are estimated within the same Difference-in-Differences framework. Upon revisiting Butts, we discovered an omission in our regression specification—the coefficient on the ring indicator was missing—and we have corrected that.

To improve clarity, we have made some modifications from line 511 to line 572:

<u>We presented the equation for the Two Way Fixed Effects model</u> in the "Basic model of Difference-in-Differences with Two Way Fixed Effects" in line 524

<u>We added a second equation</u> in the "Difference-in-Differences with Spillover and Leakage Effects" section that explicitly incorporates the ring indicator and its interaction terms in line 559.

We clarified our notation, explaining that D=1 denotes treated units (inside PAs) and therefore (1−D)×Ring captures the interaction for untreated units located in the buffer ring in line 566.

6) In the Heterogeneous Treatment Effects section you state that you will obtain an estimate of the effect of protection within each PA for each year post-intervention. Unless you are planning on conducting an event-study type analysis, which I assume you are not because it is not mentioned anywhere in the text, you will not get an estimate for each year post-protection. Your estimates will represent the average annual change in deforestation in the PA across all years post-protection.

With your study design you will be able to assess spatial heterogeneity, the different performance of different PAs, but not temporal heterogeneity. Including the regression equation for your main analysis would make this very clear.

As such, please delete the text referring to assessing changes in performance over time and instead focus on the patterns, and possible causes of spatial variation in effectiveness.

You are right that we will report the average annual change in deforestation in the PA over the entire post-protection period. However, we also plan to plot the event-study annual coefficients and confidence intervals to assess the plausibility of our findings without interpreting them at this stage. A detailed event-study analysis will be conducted in future works.

7) You need to state the data and code used in the study will be made publicly available (e.g. on Github or FigShare) after the study is complete.

Yes, you are right. We have addressed this in lines 352- 358 stating that the code will be made available on GitHub and on Software Heritage https://www.softwareheritage.org/ , and that the data will be accessible through the DataSuds portal (https://dataverse.ird.fr/).

8) I really like the idea of trying to explain the differential effectiveness of PAs according to a set of hypothesized explanatory factors but I am concerned this might be too much for a single study and may be better reserved for a follow-up.

Thank you for your suggestion. We agree that such analysis would be too much for this study. We plan to have an exploratory analysis that will be a preparation for a more detailed follow-up study.

Detailed comments:

Line 44- 46: The GFW platform uses Hansen et al., (2013) data so directly cite this data source, and delete the reference to Global Forest Watch.

We have rewritten the introduction and reformulate it by mentioning the World Resources Institute in lines 58 – 59.

Line 46 – 48: Delete the statement about how Biodiversity can play a crucial role in the developing country as this is too simplistic are currently written. Instead, you can talk about how Madagascar is one of the poorest countries in the world and it's forests play a crucial role in supporting the livelihoods of its predominantly rural populations by providing ecosystem services.

The correction has been made in lines 65 – 68

Line 49: Delete 'with the creation of Madagascar's Protected Areas System (SAPM) because otherwise it sounds like Madagascar didn't have any PAs before 2003.

It has been deleted

Line 52-53: Explain what a negative or null effect means, that these PAs did not decrease deforestation and may even have conversely increased it.

The explanation has been made in lines 84 – 85.

Lines 53- 60: I don't think you can talk about before-after comparisons without describing what they are. I also don't think you need to go into this much detail here. It is probably sufficient to say that the Wolf study showed that globally PAs established between 2002 and 2017 showed no significant effect overall, probably due to heterogeneity.

It has been revised in lines 85 -88.

Line 65-66: I don't understand this statement. Are you saying that the imagery used didn't correspond to the period 1990-2000?

It has been deleted

Line 71-72: Start this sentence with 'However…'. as it describes contrasting/contradictory results. Desbureaux showed that PAs reduced deforestation risk by 20% but this might not have reduced deforestation at the landscape-scale because deforestation was displaced into the surrounding, non-protected landscape.

It has been added in line 108.

Line 80: I'm not sure we need this much detail about the estimand they use. But please state exactly what 'modest results' means in terms of the amount of deforestation. Does that mean they found small reductions in deforestation within PAs?

It has been reformulated in lines 116 – 118

Lines 83 – 89: I recommend deleting this. These limitations described in the Harper et al., data come from the Eklund et al paper but seem to be over-emphasized compared to the original source. Also, without comparing multiple data sources to ground truth data you can't say which one is 'better'. See comment 2 below.

It has been deleted.

Line 82- 83: Cite Harper et al., here.

It has been done in line 121.

Line 94: Delete 'inaccurate data'. With these datasets it is very difficult to say which ones are 'right' or not, especially at the national scale.

It has been deleted.

Line 99: Delete 'state of the art'.

It has been deleted.

Lines 93-97: Instead of focussing on the limitations of previous studies, who did what they could with the data available at the time, focus on the novel aspects of your study: the longer time-series, the spillover analysis and particularly that you will get both average (across all PAs) and individual treatment effects. That's cool!

Thank you for your recommendation, it has been reformulated to emphasize the contribution of our work in 131 – 136.

Line 131: Rubins' causal model of potential outcomes (please add this underlined text).

It has been added in line 202.

Lines 153 – 170: Remove the table and reference to Fritz et al., as these findings refer to the whole of the African continent. Instead, try and find specific references about the leading drivers of deforestation in Madagascar. You don't need to go into much detail. You can just have one or two sentences saying something like: In Madagascar, the largest driver of

deforestation is clearance for subsistence agriculture, in some parts clearance for XXX also plays an important role. But make sure you include Madagascar-specific citations. The table has been deleted and the specific references has been added in lines 234 – 247.

Lines 199- 201: Unnecessary, you can delete this sentence.

It has been deleted.

Line 215 – 217 and Lines 220- 221. Same. Delete, this is unnecessary.

It has been deleted.

Line 230 – 235: As the study was not repeated using different PA boundary data there is no evidence that this affected the main conclusions of the study. Delete

It has been deleted.

Line 253 - 257: This paragraph is currently a bit too long and repetitive, please shorten and remove unnecessary information (e.g. that Global Forest watch was established by WRI).

It has been revised in lines 336-339.

Line 259: Just say that the data will be analyzed and processed on R. No need for the extra information.

It has been revised in line 346.

Lines 273 – 281: It is unclear how you will incorporate ecological differences between forest types into your outcome variable. Please clarify. Will you use the Hansen et al canopy cover dataset for 2000 to set a specific threshold to map forests of each type, and then mask the

deforestation data to this 2000 forest cover map? Also, see my main point about combining the Harper et al and the Hansen et al datasets to create an outcome variable.

Thank you for your comments. We have explained how we are going to use the Hansen et al. (2013)'s data and how we are going to incorporate ecological differences between simplified forest types into our analysis in lines 366 – 377. We have also followed your recommendation to mask the Hansen et al.(2013) data with the Harper et al.(2007) data as a robustness test.

Line 282: I recommend you add the paragraph explaining matching methods here, before you talk about matching variables. Please clearly explain what are the benefits of matching including specific reference to the need to control for confounding factors associated with both treatment assignment (i.e. where a PA is located) and outcomes. Failure to control for these factors can mean any differences in outcomes between the two samples might be attributable to these factors, and not the PA status at all. Matching can be used to control for confounding factors to ensure both samples are as similar as possible in characteristics which affect treatment assignment and deforestation.

It has been revised in lines 383 – 395.

Line 301: Delete 'for the nearby population' as this is often not true.

It has been deleted.

Line 310: I don't understand the difference between matching variables and control variables. Are you not matching on the control variables? Please explain.

It has been explained in lines 397 – 400 for the matching variables and in lines 430 – 435 for the control variables.

Line 333-334: I disagree with this statement, please delete. In some places forest plots closer to

rivers have a higher probability of deforestation as they are more accessible by water. Furthermore, settlements tend to be located closer to rivers also increasing the likelihood of deforestation. The reference supporting this statement is from a case study in India and should not be used.

It has been deleted.

Line 384: Changes in the evolution after the intervention.

It has been changed in line 516.

Lines 382- 389: Here you need to explain what difference-in-difference is → comparing outcomes before and after an intervention between treatment and control to assess how an intervention changed outcomes in the treated unit over time, compared to the control.

The explanation has been added in lines 511 – 514

Line 394: Replace 'fall' with 'reduction'.

It has been replaces in line 537

Line 408: Replace 'treatment unit' with 'buffer zone ring'

It has been reformulated in lines 552 – 554.

Line 411: Should be Y with subscript i and t.

The subscripts have been added in line 559.

Line 433: Reword to state that units within 20km buffer of a PA are removed from the control sample for the main analysis, and assigned as treated for the leakage/spillover analysis.

It has been reformulated in line 585 – 587.

Lines 439 – 442: Can you reword this more simply? Are you saying that for canopy cover thresholds for defining forest cover are set based on the forest type within each PA. There are only four forest types so I recommend stating explicitly here what thresholds you will set for each forest type (e.g. X% for the humid forest, Y% for the dry forest etc…).

It has been reformulated in line 593- 596. At this stage, we cannot determine the average threshold for each simplified forest type.

Line 448: 'Perform a sensitivity test for unobserved bias according to…' Please explain more clearly how unobserved bias could impact results via confounding.

The explanation has been added in lines 608-612.

Line 458: Delete 'per year'. See Main Point 4.

It has been deleted.

List of changes in the revised manuscript:

Part of the manuscript	Modifications done
Introduction	It has been rewritten to incorporate all the reviewers'comment (Line 50- 159).
Materials and Methods	• PRE-ANALYSIS PLAN OBJECTIVES<br>○ The paragraph about the pre-analysis plan objectives has been moved to the Materials and Methods. (Line 180- 198).<br>• DESIGN<br>○ The first sentence in the design section has been

	reformulated according to the reviewer's comment (Line 200).<br>• <u>OBSERVATION UNITS</u><br>◦ The table previously included in the Observation Units section has been removed, and the paragraph about the drivers of deforestation in Madagascar has been revised according to reviewer's comment. (Line 232- 256).<br>• <u>SAMPLING DATA</u><br>◦ Some paragraphs has been deleted in the Sampling Plan section according to the reviewer's comment. (Lines 276-278 ; 291-294 ; 297-300).<br>◦ We have updated the section on protected areas to include the most recent and accurate available data (Lines 304- 330). The paragraph on the forest cover has been revised in accordance with the reviewer's comment (Lines 334- 342).<br>◦ The pargraph on Data Processing Instruments has been revised to address the reviewer's comment concerning the lines of code and data availability. (Lines 344- 356).<br>• <u>EMPIRICAL ANALYSIS</u><br>◦ The paragraph on the outcome variable has been revised to address the reviewer's comment (Lines 364- 379).<br>◦ The paragraph on the matching method has been

	relocated and specific references have been added in accordance with the reviewer's comment (Lines 380-393).<br>○ The paragraph on matching variables has been revised to address reviewer's comment. (Lines 395-398).<br>○ Some reformulations have been made (Lines 412-417).<br>○ The paragraph on control variables has been revised to address the reviewer's comment (Lines 428 – 434).<br>○ This paragraph has been deleted to simplify the analysis (Lines 448 – 452).<br>○ This paragraph has been removed in line with the reviewer' s comment (Lines 453- 455).<br>• <u>ANALYSIS PLAN</u><br>○ This section has been revised to add precision in the analysis (Lines 494- 497).<br>○ This paragraph has been revised to address the reviewer's comment (Lines 507 – 515).<br>○ This equation has been added to follow the reviewer's recommendation (Lines 522- 530).<br>○ This paragraph has been revised in line with the reviewer's comment (Lines 542-555).<br>○ This equation has been revised (Lines 557 – 570).<br>○ This paragraph has been deleted to facilitate the analysis (Lines 578-582)<br>○ This section has been revised according to reviewers

	comment (Lines 583- 587).<br>○ This section has been revised in line with the reviewer's comment (Lines 591-597).<br>○ The section has been added to address the reviewer's comment (Lines 603-604).<br>○ This paragraph has been revised to address the reviewer's comment (Lines 605-610).
DISCUSSION	• <u>HETEROGENOUS TREATMENT EFFECTS/</u><br>○ Some revisions have been made to address the reviewer's comment (Lines 624-630).

We hope that these revisions meet the reviewers'expectations and we remain at your disposal for any further clarification.

Yours respectfully,

Diamondra Ramiandrisoa, on behalf of all co-authors.

Corresponding author
