# Supplementary material for: Impact of protected areas on deforestation in Madagascar from 2000 to 2023: A pre-analysis plan
Source: PLoS One. 2026 Feb 9;21(2):e0342093. doi: 10.1371/journal.pone.0342093 (PMC12885278; doi:10.1371/journal.pone.0342093)
Supplement: Attachment — Submitted filename: Response to Reviewers.docx [file pone.0342093.s003.docx]

**Impact of protected areas on deforestation in Madagascar from 2000 to 2023: A pre-analysis plan**

**RESPONSE TO EDITOR AND REVIEWERS**

Dear Dr Daniel Silva,

We are very grateful to you for agreeing to review the revised version of our manuscript: "Impact of Protected Areas on Deforestation in Madagascar from 2000 to 2023: A Pre-Analysis Plan."

We acknowledge that some aspects of the previous version could still be clarified. Therefore, we have carefully addressed each of the reviewers’ comments point by point to provide the necessary clarifications and improve the overall understanding of our pre-analysis plan. The comments and recommendations have been fully considered and are addressed below:

**Response to reviewers:**

**Reviewer #2:**

**Thank you for your courteous response to my comments. The changes you made have significantly improved the clarity of the manuscript. However, I still have a major concern about the DiD models and the assignment of treatment and control that was insufficiently addressed by the revisions. Without properly addressing these comments I cannot recommend this paper be published because I have doubts about the proposed models which have not been adequately explained.**

Thank you very much for your response and for your valuable comments, which helped us further clarify our approach and refine several key aspects of the pre-analysis plan. Regarding the methodology, we have expanded this section to remove any ambiguity and to enhance both the clarity and reproducibility of the study.

**Main point:**
**I’m sorry but I’m still very unclear on whether the spillover and leakage analysis is a separate analysis to the DiD to estimate the effect of PAs on deforestation inside PAs, shown in Line 400.
To estimate the effect of protection of a Protected Area on deforestation you need to compare outcomes within 1km^2^ grid cells inside the PA to matched control units which are unaffected by the treatment. To be sure matched control units are unaffected by the treatment they must be outside the 20km buffer zone, to ensure outcomes in these units are not biased by spillover/leakage effects. In this case your treated units come from inside the PA and the control units come from areas outside the 20km buffer zone.**

**But that means the leakage and spillover analysis and equation in Line 430 must be a separate analysis because you want to look at outcomes within this 20km buffer zone. So, given that, what do the ‘treatment’ and ‘control’ pixels in the leakage and spillover analysis represent?**

**Lines 451-453 suggest that the leakage and spillover analysis is a separate analysis comparing units within the buffer zone (assigned as ‘treated’) to more distant control units, I’m assuming outside the buffer zone. But if this is true then the notation (1 – D) x Ring is incorrect as the units you are interested in (the buffer units) will be assigned a 1 and that coefficient will be differenced out (because 1-1 = 0).**

**However, this directly contrasts with your response to my question in the last review where you said:**

**‘We clarified our notation, explaining that D=1 denotes treated units (inside PAs) and therefore (1−D)×Ring captures the interaction for untreated units located in the buffer ring in line 564.’**

**If this is correct, then you will be comparing units within protected areas (treatment = 1) to control units within the buffer zone (treatment = 0) to estimate the effect of the PA on deforestation. This is wrong because deforestation in those buffer units can be biased through spillover and leakage effects. Unless, the model controls for that?**

**This is the most important part of your analysis and it is currently not well enough explained such that it raises some red flags (concerns). Until this is clarified I cannot recommend this article is published.**

**I hope my points make sense. If not, I would urge you to ask colleagues to review the paper and see what they think. They may be able to explain my concerns.**

Thank you very much for this thoughtful comment. We understand that the distinction between the two equations may have caused some confusion, and we have clarified this point in the revised version of the manuscript.

The first equation (line 406) corresponds to the standard DiD model, which estimates the effect of protection *within* PAs. In this specification, treated units are 1km² cells located inside PAs, with control units located more than 20 km away from PA boundaries, to ensure that they are not affected by spillover or leakage effects.

The second equation in line 468, extends this canonical model following Butts (2023) to account for spatial spillovers:

$Y_{it}=\alpha+{\tau D}_{it}+\sum_{j=1}^{3} ɣ_{j}\left( 1-D_{it} \right){Ring}_{ij}+{\beta X}_{it}+\mu_{i}+\lambda_{t}+\varepsilon_{it}$

It jointly estimates:

(i) the treatment effect ($\tau$) on treated units (inside PAs); and
(ii) (ii) the spillover effects ($\gamma_{j}$) on non-treated units located within successive distance rings around PAs (with j = 1 for 0-5km, j = 2 for 5-10km, and j = 3 for 10-20km). Each $\gamma_{j}$ measures the average change in deforestation within ring j relative to control units located beyond 20 km.

For treated units ($D_{it}=1$), the spillover term is zero ($\left( 1-D_{it} \right){Ring}_{ij}=0$), yielding $\tau,$ the estimated effect of protection. For non-treated units located in the buffer, the treatment term is 0 ($D_{it}=0$), and the ring indicator is one ($\left( 1-D_{it} \right){Ring}_{ij}=1$), yielding $\gamma_{j}$, the protection spillovers or leakagefor ring j. Both treated and buffer units are compared to a common control group: the cells located beyond 20 km from any PA.

In their review of the recent advances in the state of the art for difference-in-difference methods, Roth et al. (2023) emphasize that standard DiD models rely on the Stable Unit Treatment Value Assumption (SUTVA), which is violated in the presence of spillovers. In the case of spatial spillovers, Roth et al. (2023) point to the Butts approach that we are proposing to adopt, in order to factor in these spillovers in the total effect estimation.

We have clarified this point in the manuscript and now state explicitly that the treatment and spillover effects will be reported separately besides the total effect. The concrete implementation in R will follow the procedure illustrated in the Tennessee Valley Authority replication example in Butts (2023), available at <https://github.com/kylebutts/Spatial-Spillover/blob/master/code/TVA/analysis.R>

**Specific points:**

**• Line 65: Delete ‘the country’**

It has been deleted in line 64.

**• Line72-73: I thought orphan parks were the other way around, they had legal status as a PA but no management authority? Please check and revise if I am correct?**

Thank you for your comment. You are right, paper parks refer to protected areas without effective management, while orphan parks are those lacking managers. This clarification has been incorporated in lines 70-72.

**• Lines 78- 80: Sorry, I still don’t like this sentence. It would be much better to say that X% show a negative effect, which means that they reduce deforestation, however, Y% were shown to have no effective while Z% conversely increased deforestation.**

Thank you very much for your comment. We tried to identify the percentage; however, this information is not provided in the original paper. Nevertheless, we have reformulated the sentence to: ‘‘The meta-analysis conducted by Börner et al.(2020) shows that while protected areas can reduce deforestation, they may also have no effect or even increase deforestation in some cases’’ to make it clearer in lines 76-78.

**• Line 95: To clarify consider rephrasing to: ‘… Desbureaux et al showed how PAs displaced deforestation into the surrounding landscape, partially offsetting the observed reductions in deforestation inside the PAs.’**

It has been rephrased in lines 92-94.

**• Move the explanation of matching in Lines 123-124 to Lines 116. This is a broader explanation which should be followed by a more specific explanation of the exact matching algorithm you use (Coarsened Exact matching).**

It has been moved in lines 114-116.

**• Line 166: Add ‘which influence the probability of receiving the treatment (i.e. protection) and outcomes, i.e. deforestation.**

It has been added in lines 164- 166.

**• Line 192: Change ‘higher’ to ‘lower’ resolution. While the pixel area is higher, this corresponds to lower resolution.**

It has been changed in line 191.

**• Line 226 – 229: More recent versions of the accessibility data by Nelson et al., 2019 (**[**https://www.nature.com/articles/s41597-019-0265-5**](https://www.nature.com/articles/s41597-019-0265-5)**) provide travel time estimates for different population size classes, providing data on distance to cities of** [**5,000, 15,000, 50,000**](callto:5,000,%2015,000,%2050,000) **people and higher. As accessibility here is a measure of distance to markets and demand, I recommend using this updated dataset and distance to cities with >5,000 people. This is because towns and cities with population size 5,000 – 50,000 will still exert considerable demand pressures on forest land and resources.**

Thank you very much for your suggestion. We agree with the advantages of the Nelson et al. (2019) dataset and it is the one that we initially planned to use. However, we realized that this dataset was built with 2015 as reference year, which poses an endogeneity problem and led us to favor the Uchida and Nelson (2011) dataset, as it used 2000 as reference year. Our analysis focuses on protected areas established between 2002 and 2022 and according to Ho et al. (2007), the covariates must either not be plausibly affected by the treatment or be measured prior to treatment. The construction or maintenance of roads, are likely affected by the presence of PAs (depending on the settings, to comply with conservation rules and avoid anthropic pressure, or to facilitate tourism), therefore we need accessibility data that predates our study period.

**• Lines 234 – 243: Please describe what you will do in cases of spatially overlapping PAs? For example, I can see on the WDPA data (protectedplanet.net) that Tsaratanana National Park overlaps with the Complexe des AP Ambohimirahavavy Marivorahona.**

If two PAs overlap, or if a recent PA is established within the buffer of an older one, priority is given to the older protected area in order to ensure that there is only one treatment. This clarification has been added in lines 235-238.

**• Line 272: Define what the acronym TCD is**

The acronym TCD means Tree Canopy Density. It was alread defined on line 130 and we added another definition in line 272.

**• Line 278: State and reference where you are getting these simplified forest types from**

The simplified forest types used in our study are derived from the TEOW datasets. It has been stated in line 279

**• Lines 279 – 280: I recommend saying ‘we will then apply the specific threshold for each forest type to the Hansen et al., tree canopy cover map of 2000 to create a map of baseline forest cover in 2000 tailored to each forest type. You will analyse deforestation within this 2000 forest extent.’**

It has been rephrased in lines 280-282.

**• Line 286: What polygon units will you use to define non protected areas. Protected Areas have a defined boundary but not protected areas do not.**

The polygon units representing unprotected areas correspond to 1km² grid cells located outside the boundaries of protected areas. This clarification has been added in lines 288-289.

**• Line 299: Add ‘and also influence deforestation’.**

It has been added in line 302.

**• Line 319: Why? Add something about these lands being suitable or marginal for productive uses because they are high elevation, far from markets, or have poor quality soils. Cite Joppa, Lucas N., and Alexander Pfaff. "High and far: biases in the location of protected areas."** **PloS** **one 4, no. 12 (2009): e8273.**

Thank you for your suggestion. We already cite this Joppa and Pfaff in this paragraph (it is the reference 43). As this paragraph focuses on slope and altitude, we added an explanation on lines 322-325 to specify that we refer here to the opportunity costs for resource extraction and agricultural activities. We also cite this same reference 43 (Joppa and Pfaff, 2009) in 3 other instances, among them the paragraphs referring to accessibility and population density variables.

**• Line 332-334: Delete this because I don’t think it is correct. The control variables do not ensure similarity between treatment and control groups – this is the role of the matching variables.**

It has been deleted.

**• Control Variables – are these calculated at annual resolution? Please state the time scale at which these variables are measured and included in the model**

The control variables are calculated at an annual resolution based on monthly data. The drought indicator is available from 1997 to 2023 and is computed annually with a three-year lag to capture delayed ecological responses. The cyclone exposure variable is available from 1999 to 2023 and is computed annually with a one-year lag to account for the consequences of previous cyclones. These are specified in lines 337, 347-348, 351- 353.

**• Lines 371 – 375: 30% is a high ‘acceptable’ threshold for loss of treated units. Losing treated units which cannot be matched means that your data no longer represents a population but a sample of the population. Un-matched treated units may be non-randomly distributed (e.g. they are all the highest elevation parts of a PA) which can bias the ATT if you exclude these areas. You are correct that there are trade-offs between balance and finding matches for the majority of treated units. However, I think 30% is too high a threshold for acceptable loss of treated units and would strongly recommend reducing it to 10%. You can increase it to say 20% if absolutely necessary if retaining 90% of treated observations does not produce a SMD of less than 0.2.**

Thank you very much for your constructive recommendation. We fully agree that excluding unmatched treated units may introduce bias in the estimated ATT if those units are not randomly distributed. Your reply led us to substantially revise the proposed matching procedure.

In the revised version, we have therefore tightened the maximum acceptable exclusion threshold from 30 % to 20 %, while specifying that the optimal specification that reaches a SMD ≤ 0.25 while retaining the largest possible share of treated units. In addition, for any protected area where achieving satisfactory balance requires discarding more than 10 % of treated units, we will also report an alternative estimate based on a coarser binning (SMD > 0.25) that retains at least 90 % of treated units.

We have also revised the target SMD, which was initially set at 0.2, and adopted 0.25 as an acceptable threshold, following the criterion used by Devenish et al. (2022, p. 505). Although the difference is modest, this adjustment should reduce the number of excluded treated units while remaining within the range generally considered indicative of satisfactory covariate balance.

We have also removed the requirement that all continuous variables share the same number of bins. We think that this modification will avoid unnecessary data fragmentation and better accommodates differences in the empirical distributions of covariates, such as between skewed variables (e.g., accessibility, population density) and more normally distributed ones (e.g., slope, elevation).

These revisions have been added in lines 370–394 of the revised manuscript.

**• Line 392: ‘evolve’ is not the right word here. Try ‘show the same trend in outcomes before the intervention****, meaning it is reasonable to assume that they would have continued to follow the same trend in the absence of the intervention’.**

Thank you for your recommendation. This statement has been incorporated in line 400-402.

**• Line 463: This robustness test is not to measure differences caused by spatial autocorrelation but to test the effect of the choice of scale. Please change this sentence to state this.**

Thank you for your comment, it has been changed in lines 508-509.

**List of changes in the revised manuscript:**

| Part of the manuscript | Modifications done | Lines |
| --- | --- | --- |
| Introduction | Some modifications have been made according to reviewers’ comments | Lines 46 -140 |
| Materials and Methods | - DESIGN   Some precision has been made according to reviewers ’comment in lines 165-166   - OBSERVATION UNITS   The word higher has been replaced by lower in accordance with reviewers’ comment.   - SAMPLING PLAN   Additional precision has been added regarding overlapping PAs according to the comments from the reviewers in lines 236-237.  The data regarding protected areas have been revised and corrected.   - EMPIRICAL ANALYSIS   - The acronym TCD has been defined in line 275.   - The reference used for the analysis of simplified forest types has been added .   - Some reformulations have been made.   - Details about non-protected areas have been added.   - Additional explanation has been incorporated to address reviewers’ comment.   - Some precisions have been made in the paragraph about control variables regarding reviewers’comment. - ANALYSIS PLAN   - Some modifications have been made to address the concern raised by the reviewers.   - The paragraph about the difference-in-differences has been modified to make the methodology clearer and to address the issue raised by the reviewers.   - This sentence has been corrected to address the reviewers’ comment   - Additional tests have been included in the robustness checks related to the decision algorithm of the matching | - DESIGN   Lines 164-166   - OBSERVATION UNITS   Line 192   - SAMPLING PLAN   Lines 235-237  Lines 239-246   - EMPIRICAL ANALYSIS   Lines 130, 272  Line 279  Lines 280-282  Lines 288-289  Lines 322-323  Lines 337, 347-348, 351- 353.   - ANALYSIS PLAN   Lines 370-388  Lines 397- 488  Lines 508 509  Lines 512-517 |

We sincerely hope that the revisions made will meet the reviewers’ expectations and remain at your disposal for any further clarification.

Yours respectfully,

Diamondra Ramiandrisoa, on behalf of all co-authors.

References cited in the response:

Butts, Kyle. 2023. "Difference-in-Differences Estimation with Spatial Spillovers". arXiv:2105.03737. Preprint, arXiv, June 10. <http://arxiv.org/abs/2105.03737>.

Roth J, Sant’Anna PHC, Bilinski A, Poe J. What’s trending in difference-in-differences? A synthesis of the recent econometrics literature. Journal of Econometrics. 2023;235: 2218–2244. doi:10.1016/j.jeconom.2023.03.008
