# Supplementary material for: Impact of protected areas on deforestation in Madagascar from 2000 to 2023: A pre-analysis plan
Source: PLoS One. 2026 Feb 9;21(2):e0342093. doi: 10.1371/journal.pone.0342093 (PMC12885278; doi:10.1371/journal.pone.0342093)
Supplement: Attachment — Submitted filename: Response to reviewers V2.docx [file pone.0342093.s004.docx]

**Impact of protected areas on deforestation in Madagascar from 2000 to 2023: A pre-analysis plan**

**RESPONSE TO EDITOR AND REVIEWERS**

Dear Dr Daniel Silva,

Thank you for the opportunity to submit a new revised version of our manuscript entitled: “Impact of Protected Areas on Deforestation in Madagascar from 2000 to 2023: A Pre-Analysis Plan.”

We greatly appreciate the time and effort that you and the reviewers have devoted to evaluating our work since the first submission. We are grateful to the reviewers for their insightful comments and for the decision of minor revisions.

We have incorporated the changes in accordance with the suggestions and comment provided.

Below, we present a point-by-point response to the reviewers’ comments and recommendations.

**Response to reviewers:**

**Reviewer #2:**

**Thank you. The changes you have made to the text in response my previous comments have made the analytical strategy a lot clearer and easier to understand. And I appreciate the decision algorithm you have included in the matching process to try and minimise the loss of treated units while maintaining balance. That’s a really good idea.**

**I can now recommend that this article is accepted, pending clarification of how percentage deforestation will be measured (see comment about Line 267-268). This is the only essential change.**

**I have a few additional small comments to improve the clarity of the text but these are very minor changes which should not take more than a few minutes to address.**

Thank you very much for your message and for the valuable comments and suggestions. They have been extremely helpful in improving the clarity of our study and strenghtening our analytical approach.

We also sincerely appreciate your recommendation for acceptance, it is very encouraging.

In the revised manuscript, we have carefully addressed your comments, as detailed below :

**Line 63: Change ‘beyond’ to ‘behind’**

It has been changed in line 62.

**Line 91: Is it a reduction in deforestation ‘risk’ (which implies probability) or the estimated reduction in absolute deforestation?**

Thank you for your comment. Indeed, in the Discussion and Conclusion sections, Desbureaux et al. [1] refer to a 20 % reduction in deforestation, not to deforestation risk. We therefore removed the word “risk” at line 91.

**Line 100-101: Please clarify whether this decrease was relative to the previous period, or relative to a control/counterfactual, or both?**

Thank you for highlighting that our phrasing was ambiguous. The result reported by Eklund et al. [2] stems from the comparison of two distinct cross-sectional analysis for the subsequent decades. They compared 10-years deforestation rates between matched controls and treatment pixels on the one hand for the period 1990-2000 and on the other hand for the period 2000-2010. They do not use the diachronic feature of their dataset to implement a Difference-in-Difference approach or a similar approach. What they do is observe the variation between both decade of overall estimated impact for each biome. Our phrasing" This method, like counterfactual approaches,...", could imply that their method was not counterfactual", so we replaced it by "this counterfactual method". We also rephrased the sentence "More precisely, they found that deforestation decreased from 1990-2000 to 2000-2010 in humid forest but increased in dry and spiny forests" to describe more precisely their analytical in lines 100-104.

**Line 116 – 118: is it for each variable that researchers fix ex-ante the imbalance between treated and control? If so, please add this to this sentence?**

Indeed, it is for each variable that researchers fix ex-ante the imbalance between treated and control according to Iacus et al.[3] in 2009 and Iacus et al. [4] in 2012. This clarification has been made by adding « for each variable » in line 121.

**Line 175: The matched controls won’t have exactly the same observable characteristics so please change to say something like ‘which are similar as possible in terms of observable characteristics (…) to the PAs’.**

You are right. The proposed sentence has been added in lines 177-178.

**Line 192 – 193: Forest cover, slope and elevation are measured at 0.09 ha, not 1ha. 30x30 = 900m2 = 0.09 ha**

Thank you very much. We have corrected the resolution to 0.09 ha in line 196.

**Line 237 – 239: Does this mean that the year of treatment is assigned as the year the older park was created?**

Yes, exactly. We have made it clearer by rephasing the sentence to “ In such cases, the treatment year is assigned as the year the older park is created to ensure that there is only one treatment” in lines 240-242.

**Line 267-268: Will deforestation be measured as a percentage of grid cell area or baseline forest cover?**

Thank you very much for your question. Indeed the method used to compute the outcome was not yet sufficiently clear. We will download the forest cover data using the mapme.biodiversity package, which derives these measures from Global Forest Watch data based on Hansen et al.[5] . In this package, forest cover is expressed as the percentage of each grid’s area, on a 0-100 scale (0 = no forest; 100 = completely forested).

In our study, we will start from the percentage of each grid cell covered by forest in 2000. The annual percentage of deforestation will then be computed, for each grid cell, as the share of the its area lost between year n and n+1.

For clarity, we added the following sentence in lines 274-276: “The annual percentage of deforestation will be computed for each grid cell, as the share of its forested area at year n that is lost by year n+1”.

We also replaced the word “intact” with “forested” in line 282 to ensure consistency with the Tree Canopy Density threshold approach.

**Line 287: Change to ‘In this study, the treated units are 1km grid cells within PAs and the control units are grid cells in unprotected areas outside the boundaries of any protected area.’**

It has been changed in lines 293-295.

**Line 328: Change ‘area’ to ‘1km grid cell’**

It has been changed to 1 km² grid cell in lines 332-333.

**Line 362: Are units assigned to bins based on the coarsened values of all of their covariates? If so, add ‘all’ to this sentence**

Yes, the units are assigned to bins based on the coarsened values of all their covariates. The word « all » has been added to the sentence in line 367.

**Line 364 – 365: You can delete this sentence because it is repetitive**

It has been deleted.

**Line 371: Add ‘between treatment and matched controls’ after ‘Standardised Mean Difference’.**

It has been added in 375.

**Line 430: Add the qualifier ‘potential’ before ‘occurrence of spillover and leakage effects’.**

It has been added in line 435.

**Line 435: Add ‘from the PA’ at the end of this sentence.**

It has been added in line 440.

**Line 436: Change ‘the total effect on the treated units’ to ‘the total effect of protection on treated units within the PA’.**

It has been changed in lines 441-442.

**Referecences cited in the response**

1.	Desbureaux S, Aubert S, Brimont L, Karsenty A, Lohanivo AC, Rakotondrabe M, et al. The impact of Protected Areas on Deforestation? An Exploration of the Economic and Political Channels for Madagascar’s Rainforests (2001-12). Working Papers. HAL; 2015 July. Report No.: hal-01176860. Available: https://ideas.repec.org/p/hal/wpaper/hal-01176860.html

2.	Eklund J, Blanchet FG, Nyman J, Rocha R, Virtanen T, Cabeza M. Contrasting spatial and temporal trends of protected area effectiveness in mitigating deforestation in Madagascar. Biological Conservation. 2016;203: 290–297.

3.	Iacus SM, King G, Porro G. **cem** : Software for Coarsened Exact Matching. J Stat Soft. 2009;30. doi:10.18637/jss.v030.i09

4.	Iacus SM, King G, Porro G. Causal inference without balance checking: Coarsened exact matching. Political analysis. 2012;20: 1–24.

5.	Hansen MC, Potapov PV, Moore R, Hancher M, Turubanova SA, Tyukavina A, et al. High-resolution global maps of 21st-century forest cover change. Science. 2013;342: 850–853. Following the revisions made to the manuscript, we provide below the list of the references that were removed and those that were added compared with the first version. These updates were implemented in response to the reviewers’suggestions to improve the internal consistency of the study and strengthen the scientific rigor of the manuscript.

**References removed compared with the first version :**

- Cardinale BJ, Duffy JE, Gonzalez A, Hooper DU, Perrings C, Venail P, et al. Biodiversity loss and its impact on humanity. Nature. 2012;486: 59–67. doi:10.1038/nature11148
- Méral P, Froger G, Andriamahefazafy F, Rabearisoa A. Chapitre 5. Le financement des aires protégées à Madagascar : de nouvelles modalités. In: Aubertin C, Rodary E, editors. Aires protégées, espaces durables ? Marseille: IRD Éditions; 2013. pp. 135–155. doi:10.4000/books.irdeditions.5677
- Ribas LG dos S, Pressey RL, Loyola R, Bini LM. A global comparative analysis of impact evaluation methods in estimating the effectiveness of protected areas. Biological Conservation. 2020;246: 108595. doi:10.1016/j.biocon.2020.108595
- Hansen MC, Loveland TR. A review of large area monitoring of land cover change using Landsat data. Remote Sensing of Environment. 2012;122: 66–74. doi:10.1016/j.rse.2011.08.024
- Fritz S, Laso Bayas JC, See L, Schepaschenko D, Hofhansl F, Jung M, et al. A Continental Assessment of the Drivers of Tropical Deforestation With a Focus on Protected Areas. Frontiers in Conservation Science. 2022;3. Available: https://www.frontiersin.org/article/10.3389/fcosc.2022.830248
- Danko D M. Proceedings of the Eleventh Annual ESRI User Conference. Environmental Systems Research Institute; 1991.
- Knapp KR, Kruk MC, Levinson DH, Diamond HJ, Neumann CJ. The International Best Track Archive for Climate Stewardship (IBTrACS): Unifying Tropical Cyclone Data. Bulletin of the American Meteorological Society. 2010;91: 363–376. doi:10.1175/2009BAMS2755.1
- Gahtan J, Knapp KR, Schreck CJI, Diamond HJ, Kossin JP, Kruk MC. International Best Track Archive for Climate Stewardship (IBTrACS) Project, Version 4.01. 2024 [cited 21 Nov 2024]. Available: https://www.ncei.noaa.gov/access/metadata/landing-page/bin/iso?id=gov.noaa.ncdc:C01552
- Eklund J, Jones JP, Räsänen M, Geldmann J, Jokinen A-P, Pellegrini A, et al. Elevated fires during COVID-19 lockdown and the vulnerability of protected areas. Nature Sustainability. 2022;5: 603–609.
- Andrianambinina FOD, Waeber PO, Schuurman D, Lowry PP, Wilmé L. Clarification on protected area management efforts in Madagascar during periods of heightened uncertainty and instability. Madagascar Conservation & Development. 2022;17: 25–28.
- Goodman SM, Raherilalao MJ, Wohlhauser S, Rabenandrasana JCN, Rakotondratsimba HM, Andriamialisoa F, et al. Les aires protégées terrestres de Madagascar: leur histoire, description et biote. Association Vahatra; 2018. Available: https://zoboko.com/publisher/association-vahatra-in-antananarivo
- R Core Team. R: A Language and Environment for Statistical Computing. Vienna, Austria: R Foundation for Statistical Computing; 2021. Available: https://www.R-project.org/
- Jones JPG, Mandimbiniaina R, Kelly R, Ranjatson P, Rakotojoelina B, Schreckenberg K, et al. Human migration to the forest frontier: Implications for land use change and conservation management. Geo: Geography and Environment. 2018;5: e00050. doi:10.1002/geo2.50
- Andam KS, Ferraro PJ, Pfaff A, Sanchez-Azofeifa GA, Robalino JA. Measuring the effectiveness of protected area networks in reducing deforestation. Proceedings of the National Academy of Sciences. 2008;105: 16089–16094. doi:10.1073/pnas.0800437105
- Saha S, Saha M, Mukherjee K, Arabameri A, Ngo PTT, Paul GC. Predicting the deforestation probability using the binary logistic regression, random forest, ensemble rotational forest, REPTree: A case study at the Gumani River Basin, India. Science of The Total Environment. 2020;730: 139197. doi:10.1016/j.scitotenv.2020.139197
- Ferraro PJ, Hanauer MM, Sims KRE. Conditions associated with protected area success in conservation and poverty reduction. Proceedings of the National Academy of Sciences. 2011;108: 13913–13918. doi:10.1073/pnas.1011529108

**References added compared with the first version :**

- Achard F, DeFries R, Eva H, Hansen M, Mayaux P, Stibig H-J. Pan-tropical monitoring of deforestation. Environ Res Lett. 2007;2: 045022. doi:10.1088/1748-9326/2/4/045022
- Tabor K, Hewson J, Tien H, González-Roglich M, Hole D, Williams JW. Tropical Protected Areas Under Increasing Threats from Climate Change and Deforestation. Land. 2018;7: 90. doi:10.3390/land7030090
- Vieilledent G, Grinand C, Vaudry R. Forecasting deforestation and carbon emissions in tropical developing countries facing demographic expansion: a case study in M adagascar. Ecology and Evolution. 2013;3: 1702–1716. doi:10.1002/ece3.550
- Andam KS, Ferraro PJ, Pfaff A, Sanchez-Azofeifa GA, Robalino JA. Measuring the effectiveness of protected area networks in reducing deforestation. Proceedings of the National Academy of Sciences. 2008;105: 16089–16094. doi:10.1073/pnas.0800437105
- Ma B, Zhang Y, Hou Y, Wen Y. Do Protected Areas Matter? A Systematic Review of the Social and Ecological Impacts of the Establishment of Protected Areas. IJERPH. 2020;17: 7259. doi:10.3390/ijerph17197259
- Myers N. Threatened biotas: “Hot spots” in tropical forests. Environmentalist. 1988;8: 187–208. doi:10.1007/BF02240252
- World Bank. Évaluation de la pauvreté à Madagascar: Naviguer sur deux décennies de pauvreté élevée et tracer la voie du changement. In: World Bank [Internet]. 2024 [cited 8 Oct 2024]. Available: https://www.banquemondiale.org/fr/country/madagascar/publication/madagascar-afe-poverty-assessment-navigating-two-decades-of-high-poverty-and-charting-a-course-for-change
- Gardner CJ, Nicoll ME, Birkinshaw C, Harris A, Lewis RE, Rakotomalala D, et al. The rapid expansion of Madagascar’s protected area system. Biological Conservation. 2018;220: 29–36. doi:10.1016/j.biocon.2018.02.011
- Iacus SM, King G, Porro G. Causal inference without balance checking: Coarsened exact matching. Political analysis. 2012;20: 1–24.
- Iacus SM, King G, Porro G. Multivariate Matching Methods That Are Monotonic Imbalance Bounding. Journal of the American Statistical Association. 2011;106: 345–361. doi:10.1198/jasa.2011.tm09599
- Roth J, Sant’Anna PHC, Bilinski A, Poe J. What’s trending in difference-in-differences? A synthesis of the recent econometrics literature. Journal of Econometrics. 2023;235: 2218–2244. doi:10.1016/j.jeconom.2023.03.008
- Butts K, Gardner J. {did2s}: Two-Stage Difference-in-Differences. The R Journal. 2022;14: 162–173. doi:10.32614/RJ-2022-048
- Callaway B, Sant’Anna PHC. Difference-in-Differences With Multiple Time Periods and an Application on the Minimum Wage and Employment. SSRN Journal. 2018 [cited 15 July 2024]. doi:10.2139/ssrn.3148250
- Jones JPG, Rakotonarivo OS, Razafimanahaka JH. Forest Conservation in Madagascar: Past, Present, and Future. 2021.
- Scales IR. The drivers of deforestation and the complexity of land use in Madagascar. Conservation and environmental management in Madagascar. Routledge; 2014. pp. 129–150.
- Stuart EA. Matching methods for causal inference: A review and a look forward. Stat Sci. 2010;25: 1–21. doi:10.1214/09-STS313
- Joppa L, Pfaff A. Reassessing the forest impacts of protection: The challenge of nonrandom location and a corrective method. Annals of the New York Academy of Sciences. 2010;1185: 135–149. doi:10.1111/j.1749-6632.2009.05162.x
- Angrist JD, Pischke J-S. Mostly Harmless Econometrics: An Empiricist’s Companion. 1st edition. Princeton, New Jersey Oxford: Princeton University Press; 2009.
- Baker A, Callaway B, Cunningham S, Goodman-Bacon A, Sant’Anna PHC. Difference-in-Differences Designs: A Practitioner’s Guide. arXiv; 2025. doi:10.48550/arXiv.2503.13323
- Callaway B, Sant’Anna PHC. Difference-in-Differences with multiple time periods. Journal of Econometrics. 2021;225: 200–230. doi:10.1016/j.jeconom.2020.12.001
- Abadie A. Semiparametric Difference-in-Differences Estimators. The Review of Economic Studies. 2005;72: 1–19. doi:10.1111/0034-6527.00321
- Desbureaux S. Subjective modeling choices and the robustness of impact evaluations in conservation science. Conservation Biology. 2021;35: 1615–1626. doi:10.1111/cobi.13728
- Rosenbaum PR. Sensitivity Analysis for m‐Estimates, Tests, and Confidence Intervals in Matched Observational Studies. Biometrics. 2007;63: 456–464. doi:10.1111/j.1541-0420.2006.00717.x

We also confirm that none of the references cited in the current version of the manuscript has been retracted from the scientific literature.

We sincerely hope that the revisions made will meet the reviewers’ expectations and remain at your disposal for any further clarification.

Yours respectfully,

Diamondra Ramiandrisoa, on behalf of all co-authors.
